# Complexity of Pharmacologic Pain Control—The Allegory of the Pharmacogenetic Cave

**DOI:** 10.1001/jamanetworkopen.2025.58196

**Published:** 2026-02-02

**Authors:** Andrew A. Monte

**Affiliations:** Rocky Mountain Poison & Drug Safety, Denver Health & Hospital Authority, Denver, Colorado; University of Colorado School of Medicine, Aurora.

In this study, Cavallari et al^1^ report the findings of a pharmacogenetic-guided pain control trial conducted as part of A Depression and Opioid Pragmatic Trial in Pharmacogenetics (ADOPT PGx). Cavallari et al^1^ found that genotyping prior to major surgical procedures decreased prescribing of hydrocodone but did not improve the primary composite outcome of pain control and overall opioid use. The study by Cavallari et al^1^ had numerous strengths that move the field forward in important ways. To my knowledge, it is the largest clinical trial of genotype-guided pain control ever performed; of 1602 participants enrolled, 351 had actionable intermediate or poor *CYP2D6* metabolizer (IM/PM) genotypes, of whom 176 received pharmacogenetic prescribing guidance. Cavallari et al^1^ excluded patients receiving chronic opioid therapy, which minimized the impact of preexisting tolerance. The pragmatic trial design allowed for assessment of numerous surgical postoperative pain indications, the randomization distributed comorbidities equally between groups, and Cavallari et al^1^ assessed several *CYP2D6*-dependent opioids (codeine, tramadol and hydrocodone). Additionally, the design allowed for contextual assessment of pharmacogenetic-guided opioid treatment in the setting of other nonopioid pain control practices.

Cavallari et al^1^ found that *CYP2D6* genotyping alone did not improve pertinent clinical outcomes of pain control and cumulative opioid use, but the pragmatic design leaves clinicians with several unanswered questions. Most notably, prescribing was not protocol-driven, which resulted in more than one-third of participants in both groups receiving oxycodone, which likely provided similar pain control across groups and is not significantly affected by the *CYP2D6* genotype. Hydromorphone was appropriately prescribed in 39% in the pharmacogenetic-guided group and 3% in the control group; this is expected, as hydrocodone cannot be efficiently metabolized to the active hydromorphone metabolite by those with IM/PM *CYP2D6* metabolizer phenotypes. However, it has important implications on the opioid milligram morphine equivalent (MME) analyses. The MMEs of hydromorphone 2-mg tablets, oxycodone 5-mg tablets, and hydrocodone 5-mg tablets are 10, 7.5, and 5, respectively. Because the substituted analgesics in patients with IM/PM have higher MMEs and prescribers aim for adequate pain control regardless of genotype, the design was unlikely to find a significant difference in cumulative opioid use or pain intensity scores. These additional opioid prescriptions complicate the interpretation of opioid utilization and pain control outcomes.

The pragmatic design of the study by Cavallari et al^1^ led to 64% of participants in the pharmacogenetic-guided group receiving prescribing concordant with their genotype, which was higher than the 27% concordance in the control group, yet also means the intervention did not reach more than one-third of eligible enrolled patients in the pharmacogenetic-guided group. However, in a subanalysis that eliminated the nonconcordant prescribing group, there was still no trend toward improved pain outcomes observed in the pharmacogenetic-guided group. The lack of pharmacogenetic concordant-prescribing may represent a gap in clinician education about the benefit of adherence to pharmacogenetic guidance or clinically justifiable reasons for nonconcordant prescribing, allowed under the pragmatic design. Education of both patients and clinicians could improve concordance of pharmacogenetic guided prescribing, but the outcome would not be likely to change. Paired with the lack of standardized prescribing, it remains difficult to clearly determine what effect pharmacogenetic-concordant prescribing had on the primary outcome, since clinicians are likely to have substituted oxycodone and hydromorphone analgesics for clinically justified reasons.

Genotyping of some variants with little biologic redundancy has identified some well-defined simple clinical phenotypes. Benefits of genotyping have been demonstrated for *TPMT*-azothioprine, *DPYD*-capecitabine, *HLA-B*-abacavir, and *CYP2C79*-clopidogrol, among the 34 Clinical Pharmacogenetics Implementation Consortium (CPIC) tier 1A drug-gene pairs.^2^ However, pain control is a complex phenotype, mediated by numerous biologic pathways controlled by myriad genes. Several other genes have been hypothesized to contribute to variability in pharmacologic pain control. Genes such as *COMT* and *OPRMI* are hypothesized to contribute but do not have the clinical evidence to support the CPIC designation as actionable.^3^ Variability in these genes is common, but the clinical utility of their genotyping has never been assessed in a well-controlled study with *CYP2D6* or other candidate genes. There remains a very real possibility that functional variants in these, or some of the more than 30 000 unassessed genes in the human genome paired with *CYP2D6,* are associated with clinical response. In fact, we are only beginning to understand the biologic complexity, let alone the social and behavioral complexity, of pharmacologic pain control. We may eventually find that polygenic assessments of genes in clinically complex pathways are necessary to effectively alter prescribing for complex phenotypes.

Most clinical phenotypes are not simple to identify. This trial by Cavallari et al^1^ again demonstrates the difficulty in proving improved outcomes from pharmacogenetic-guided prescribing for complex clinical conditions mediated by numerous genes. Similar negative results were observed in 2 of 3 trials^4-6^ that assessed pharmacogenetic guided coumarin and warfarin treatment, which did not improve time within the target international normalized ratio (INR) range. Like pain control, INR is dependent on many individual patient factors, including diet, concomitant medications, and numerous genes. Another pragmatic pharmacogenetic trial conducted by Swen et al^7^ demonstrated a 30% reduction in adverse events when a panel of 12 genes was paired with a point-of-care pharmacogenetic educational program.^7^ This composite outcome was less modifiable by preemptive clinical intervention; the occurrence of an adverse event was the outcome, rather than control of downstream symptoms. It is also possible clinical benefit was more likely in the trial by Swen et al^7^ due to assessment of more genes and the presence of fewer outcome confounders. The contrast between these pragmatic designs should guide future investigators toward broader genotyping strategies and choice of minimally confounded outcomes.

Although pain control and opioid utilization were unchanged by preemptive pharmacogenetic treatment, Cavallari et al^1^ demonstrated that genotyping has utility to minimize ineffective hydrocodone prescribing. Placing these results in context, pharmacogenetics is but one of many variables prescribers use to choose the right drugfor the individual patient with complex clinical symptoms. Data from a representative national survey demonstrated that only 6.6% of patients in the US have undergone pharmacogenetic treatment and only 32.2% desired pharmacogenetic testing.^8^ This highlights the need for education of clinicians about the appropriate use of pharmacogenetics.

The study by Cavallari et al^1^ suggests that complex conditions require complex polygenic testing strategies and strict control of confounders to determine clinical utility. Uptake of pharmacogenetics into regular clinical practice requires a holistic assessment of the genotype and phenotype paired with targeted education for both patients and practitioners. Like Plato's allegory of the cave, clinicians must not be misled by a minimal shadow of utility when there is such little light in the cave. Pharmacogenetics has utility, but more work is needed to fully assess their role in clinically complex phenotypes.
